# Plasma Metabolomics Reveal Alterations of Sphingo- and Glycerophospholipid Levels in Non-Diabetic Carriers of the Transcription Factor 7-Like 2 Polymorphism rs7903146

**DOI:** 10.1371/journal.pone.0078430

**Published:** 2013-10-24

**Authors:** Cornelia Then, Simone Wahl, Anna Kirchhofer, Harald Grallert, Susanne Krug, Gabi Kastenmüller, Werner Römisch-Margl, Melina Claussnitzer, Thomas Illig, Margit Heier, Christa Meisinger, Jerzy Adamski, Barbara Thorand, Cornelia Huth, Annette Peters, Cornelia Prehn, Ina Heukamp, Helmut Laumen, Andreas Lechner, Hans Hauner, Jochen Seissler

**Affiliations:** 1 Medizinische Klinik und Poliklinik IV, Diabetes Zentrum - Campus Innenstadt, Klinikum der Universität München, Munich, Germany; 2 Clinical Cooperation Group Diabetes, Ludwig-Maximilians-Universität München and Helmholtz Zentrum München, Munich, Germany; 3 Research Unit of Molecular Epidemiology, German Research Center for Environmental Health, Neuherberg, Germany; 4 Else-Kroener-Fresenius-Centre for Nutritional Medicine, ZIEL - Research Centre for Nutrition and Food Sciences, Technical University München, Freising-Weihenstephan, Germany; 5 Clinical Cooperation Group Nutrigenomics and Type 2 Diabetes, Technical University München and Helmholtz Zentrum München, Munich, Germany; 6 Institute of Bioinformatics and Systems Biology, Helmholtz Zentrum München, Neuherberg, Germany; 7 Hannover Unified Biobank, Hannover Medical School, Hannover, Germany; 8 Institute of Epidemiology II, Helmholtz Zentrum München – German Research Center for Environmental Health (GmbH), Neuherberg, Germany; 9 German Center for Diabetes Research (DZD), Neuherberg, Germany; 10 Institute of Experimental Genetics, Genome Analysis Center, Helmholtz Zentrum München, Neuherberg, Germany.; National Research Council of Italy, Italy

## Abstract

**Aims/Hypothesis:**

Polymorphisms in the transcription factor 7-like 2 (TCF7L2) gene have been shown to display a powerful association with type 2 diabetes. The aim of the present study was to evaluate metabolic alterations in carriers of a common TCF7L2 risk variant.

**Methods:**

Seventeen non-diabetic subjects carrying the T risk allele at the rs7903146 TCF7L2 locus and 24 subjects carrying no risk allele were submitted to intravenous glucose tolerance test and euglycemic-hyperinsulinemic clamp. Plasma samples were analysed for concentrations of 163 metabolites through targeted mass spectrometry.

**Results:**

TCF7L2 risk allele carriers had a reduced first-phase insulin response and normal insulin sensitivity. Under fasting conditions, carriers of TCF7L2 rs7903146 exhibited a non-significant increase of plasma sphingomyelins (SMs), phosphatidylcholines (PCs) and lysophosphatidylcholines (lysoPCs) species. A significant genotype effect was detected in response to challenge tests in 6 SMs (C16:0, C16:1, C18:0, C18:1, C24:0, C24:1), 5 hydroxy-SMs (C14:1, C16:1, C22:1, C22:2, C24:1), 4 lysoPCs (C14:0, C16:0, C16:1, C17:0), 3 diacyl-PCs (C28:1, C36:6, C40:4) and 4 long-chain acyl-alkyl-PCs (C40:2, C40:5, C44:5, C44:6).

**Discussion:**

Plasma metabolomic profiling identified alterations of phospholipid metabolism in response to challenge tests in subjects with TCF7L2 rs7903146 genotype. This may reflect a genotype-mediated link to early metabolic abnormalities prior to the development of disturbed glucose tolerance.

## Introduction

Type 2 diabetes mellitus (T2D) is a multifactorial disease resulting from a complex interaction between environment, adverse health behaviour and genetic risk factors, which may affect β-cell function and/or insulin resistance. Genome-wide association studies including thousands of cases and controls described an association of 56 single nucleotide polymorphisms (SNPs) with T2D susceptibility [1]. The contribution of most gene polymorphisms appears to be small. However, certain loci have substantial impact on the risk of T2D. Variants in the transcription factor 7-like 2 (TCF7L2) locus at 10q25.2 have been identified as the strongest common genetic risk factors for T2D in Caucasians [1,2]. TCF7L2 SNPs primarily affect insulin secretion and hepatic insulin sensitivity [3,4]. A reduced GLP-1-induced insulin secretion in response to oral glucose was detected in TCF7L2 risk allele carriers [3,5–7]. TCF7L2 SNPs were shown to affect GLP-1 responsiveness of β-cells [3,7], indicating an impaired incretin effect as one of the factors contributing to decreased insulin secretion in TCF7L2 risk allele carriers. Other studies demonstrated that TCF7L2 has an important role for vital functions in islet cells [8]. Recently, it has been shown that tissue-specific alternative splicing patterns of TCF7L2 mRNA variants determine insulin sensitivity of adipose tissue and are involved in the regulation of hepatic gluconeogenesis [9–11]. 

 However, the underlying molecular and cellular mechanisms of progression of insulin secretion deficiency and other potential metabolic alterations related to TCF7L2 SNPs are incompletely understood.

Metabolomics approaches have been successfully used to distinguish normal glucose tolerant probands and patients with impaired glucose tolerance or T2D [12–15]. Previous work focused mainly on the relationship of metabolomic alterations and insulin resistance and results are consistent with effects explained by impaired insulin action, such as diminished plasma amino acids due to increased amino acid oxidation [12] or inadequate suppression of lipolysis [16]. 

Prediction and prevention of T2D would benefit from a better understanding of the pathophysiological mechanisms involved in prediabetes and the identification of metabolic alterations associated with diabetes risk in a state when glucose homeostasis is still normal. Therefore, in the present study, we recruited non-diabetic probands carrying the TCF7L2 polymorphism rs7903146 and compared fasting plasma metabolite levels and changes in defined metabolite concentrations in response to challenge tests.

## Methods

### Study participants

Forty-one male subjects of western European descent were recruited from the population-based KORA (Cooperative Health Research in the Region of Augsburg, southern Germany) cohort [17]. All study participants gave written informed consent and the study was approved by the Ethics Committee of the Bavarian Medical Association. Eight participants were homozygous (TT) and nine heterozygous (CT) carriers of the TCF7L2 rs7903146 allele, and 24 participants carrying no risk allele (CC) with similar age and BMI served as control subjects. Genotyping of TCF7L2 was performed as described previously [18]. 

39 subjects had normal glucose tolerance (75 g oral glucose tolerance test [oGTT]), one control subject had disturbed glucose tolerance and in one carrier of the TCF7L2 rs7903146 allele with normal fasting glucose and normal HbA1c, the 2 h-glucose level was missing. Plasma samples from oGTT were not available in many probands. Probands did not take any medication known to affect insulin sensitivity or secretion. Participants underwent standard procedures including physical examination, assessment of medical history, measurement of blood pressure, and anthropometric measurements of weight, height and waist circumference. Neither the participant nor any of the attending physicians or assistants conducting the intravenous glucose tolerance test (ivGTT) and the euglycemic-hyperinsulinemic (EH) clamp knew the genotype of the probands at the time of the test procedure. 

### Metabolic challenge tests

All metabolic challenge tests were carried out between 8:00 and 9:00 a.m. after a 10 to 12 hour fasting period and after baseline blood samples were obtained. Participants underwent an intravenous glucose tolerance test (ivGTT) receiving an i.v. bolus of 0.33 g glucose/kg body weight of a 50 % (vol/vol) glucose solution within two minutes. Venous blood samples from the opposite arm were obtained at 1, 3, 5, 10, 15, 25 and 35 minutes for determination of insulin and proinsulin concentrations. The sample taken at 35 minutes (t35) was also kept for metabolomics measurement. Subsequently, the EH clamp was initiated by an insulin bolus followed by a continuous insulin infusion (1.05 mIU/kg/hour of short-acting human insulin) and a variable infusion of a 20 % glucose infusion to maintain the plasma glucose concentration at 80 mg/dl for 120 minutes. Blood samples for determination of plasma glucose were obtained at 6 minute intervals throughout the clamp and measured using a bedside glucose analyser (Super-GL Ambulance, HITADO, Möhnesee, Germany). 45 min after steady state conditions were reached (about 3-4 hours after the start of the ivGTT), venous blood samples were drawn for metabolomics measurement (t240).

### Laboratory measurements

All blood samples were immediately cooled to 4°C and centrifuged at 3000 g for 10 min. Aliquots of plasma samples were stored at -80°C until assayed. Plasma glucose levels were assessed using the hexokinase method (GLU Flex, Dade Behring, Marburg, Germany). Total cholesterol concentrations, high-density lipoprotein (HDL) and low-density lipoprotein (LDL) levels were measured with enzymatic methods (CHOD-PAP, Dade Behring). Triglycerides were measured by an enzymatic color test (GPO-PAP-method, TGL Flex, Dade Behring). Serum creatinine concentrations were assessed with a modified Jaffe test (Krea Flex, Dade Behring). High sensitive C-reactive protein (hsCRP) was determined by IRMA (Dade Behring). HbA1c was measured using the HPLC method. Plasma proinsulin and insulin concentrations were quantified with ELISA Kits (LINCO research, St. Charles, USA) as described recently [19,20]. Serum C-peptide was determined with the radioimmunoassay from Radim Diagnostics (Pomezia, Italy). Total levels of non-esterified free fatty acids (NEFAs) were measured using the Wako NEFA-HR(2) test (Wako Chemicals GmbH, Neuss, Germany). 

### Targeted *Metabolomics*

Plasma samples from three different time points were subjected to metabolomics measurement (t0, fasting sample; t35, post-ivGTT; t240, clamp-steady state). Metabolite concentrations were determined using the targeted metabolomics kit Absolute*IDQ* ^TM^ p150 (Biocrates Life Sciences AG, Innsbruck, Austria) applying mass spectrometric analysis as described previously [21]. The metabolomics data set contains hexose, 14 amino acids, free carnitine (C0), 40 acylcarnitines, hydroxylacylcarnitines and dicarboxylacylcarnitines, 15 sphingomyelins, 77 phosphatidylcholines, and 15 lysophosphatidylcholines (complete list of the analysed metabolites is shown in Table S1). Potential plate effects were addressed by multiplying metabolite concentrations by a metabolite- and plate-specific correction factor. This factor was calculated as the geometric mean of the six plate-specific geometric means of metabolite concentrations divided by the respective plate-specific geometric mean.

Furthermore, metabolites that did not meet one of the following criteria were excluded: (1) The coefficient of variation (CV) of metabolite concentrations in the five reference samples was ≥ 0.25 for all of the plates; (2) Kendall's correlation p-value was ≥ 0.1 for correlation between the sample and the reference sample means of the different plates; (3) less than 30 % concentration values were exactly zero for this metabolite. 37 metabolites were excluded, leaving 126 for analysis (Table S1). In addition, 45 metabolite sums that were assumed to indicate a certain metabolic state or process were calculated, as proposed in the Biocrates MetaDis*IDQ* ^TM^ kit manual. 

### Calculations and statistical analysis

The area under the curve (AUC) of plasma insulin during the first phase of the ivGTT was calculated according to the trapezoid method as 0.5*[0.5*c_0_(insulin) +c_1_(insulin+c_3_(insulin)+c_5_(insulin)+c_10_(insulin)] with c_x_(insulin) denoting the insulin concentration at x minutes after ivGTT. The AUC of the second-phase insulin response (time points 10, 15, 25 and 35 minutes after the glucose challenge) and the glucose response were calculated accordingly. First-phase insulin response (FPIR) was calculated as the sum of the plasma insulin values at time points 1 and 3 minutes. Clamp-derived insulin sensitivity index (ISI) was calculated as glucose infusion rate per kg body weight necessary to maintain euglycemia during the last 45 minutes of the clamp steady state per unit of plasma insulin concentration. Proinsulin conversion was estimated as proinsulin divided by insulin concentration at the indicated time points. 

 Differences in baseline anthropometric and clinical data between the two genotype groups were assessed using Mann-Whitney U tests. Distributions of the metabolite concentrations and sums, henceforth referred to as metabolite traits, were assessed using quantile-quantile-plots and most were considered to be of log-normal shape. Consequently, log-transformed metabolite traits were used for all models. Genotype and challenge effects on metabolite traits were investigated with linear mixed-effects models using the R package nlme, version 3.1-103 [22]. Specifically, for each of the metabolite traits, we included genotype, coded as 1 (risk allele carriers) and 0 (non-carriers), corresponding to a dominant genetic model, two measurement time indicators (t35 and t240, with fasting measurement as reference category) and the interaction of these with genotype to also explore the modification of challenge effects by genotype. As potential confounders, mean-centered BMI, age, and the interactions of both with measurement time, were included in all models, and random intercepts were included per each measurement time point. Restricted maximum likelihood estimation was used. P-values of genotype main effects and of genotype x measurement time interactions were subjected to correction for multiple testing using the Benjamini-Hochberg procedure [23]. All calculations concerning metabolomics analysis were performed using the statistical environment R, version 2.14.2.

## Results

### Baseline characteristics

The baseline characteristics of the probands are given in Table 1. There were no statistically significant differences in anthropometric parameters, age, family history of diabetes, waist circumference, systolic and diastolic blood pressure, plasma triglycerides, total cholesterol, LDL-cholesterol, HDL-cholesterol, creatinine, CRP and HbA1c values between TCF7L2 risk allele carriers and controls. Fasting glucose, insulin, proinsulin, and C-peptide levels were also similar in both study groups.

**Table 1:** Baseline characteristics of the study population.

	**CC genotype (n = 24)**	**CT/TT genotype (n = 17)**	**p-value**
**height** (cm)	179.7 (7.5), [180.0]	177.6 (6.3), [177.0]	0.58
**weight** (kg)	87.4 (8.9), [85.5]	85.3 (11.8), [84.5]	0.55
**BMI** (kg/m^2^)	27.1 (2.6), [26.9]	27.0 (2.7), [25.5]	0.74
**waist circumference** (cm)	99.8 (7.5), [100.5]	99.9 (9.6), [96.0]	0.75
**age** (years)	51 (10.4), [50.5]	58 (10.1), [60.0]	0.07
**systolic blood pressure** (mmHg)	140 (18.2), [136]	142 (14.2), [140]	0.49
**diastolic blood pressure** (mmHg)	83 (11.1), [83]	85 (10.8), [85]	0.57
**creatinine** (mg/dl)	0.9 (0.1), [0.9]	0.9 (0.2), [1.0]	0.25
**total cholesterol** (mg/dl)	201.1 (28.0), [202.5]	206.6 (34.2), [205.0]	0.82
**LDL-cholesterol** (mg/dl)	119.5 (31.8), [122.5]	124 (25.4), [123.0]	0.58
**HDL-cholesterol** (mg/dl)	53.0 (14), [51.5]	61.0 (19.3), [60.5]	0.31
**triglycerides** (mg/dl)	143.3 (75.8), [126.0]	114.0 (50.7), [102.0]	0.38
**CRP** (mg/dl)	0.30 (0.6), [0.1]	0.22 (0.2), [0.1]	0.70
**TSH** (µU/ml)	1.6 (0.8), [1.5]	1.9 (1.1), [2.0]	0.55
**HbA1(c**) (%)	5.5 (0.2), [5.6]	5.7 (0.3), [5.6]	0.29
**Fasting glucose** (mg/dl)	96.8 (9.7), [97.5]	97.9 (10.9), [100.0]	0.73
**2 h glucose (oGTT**)	94.4 (29.7), [94.0]	93.5 (28.5), [90.8]	0.99
**Insulin** (pmol/l)	45.9 (18.6), [43.2]	37.5 (22.0), [31.7]	0.57
**Proinsulin** (pmol/l)	3.9 (4.6), [2.3]	3.0 (2.6), [1.9]	0.30
**C-peptide** (ng/ml)	2.8 (1.6), [2.4]	2.5 (1.1), [2.0]	0.76
**NEFAs** (mmol/l)	0.6 (0.4), [0.5]	0.55 (0.3), [0.5]	0.47

Data are given as mean (standard deviation), [median]. Given are the crude p values before correction for multiple testing.

### Genotype effects in the fasting state

Fasting results in β-oxidation of fatty acids and gluconeogenesis from amino acids. As expected, we observed high plasma concentrations of non-esterified fatty acids (NEFAs), free short chain acylcarnitines (C2 and C3) and gluconeogenic amino acids (sum of glycine and serine). The sum of branched-chain amino acids (leucin, isoleucine, valine) did not differ between study groups (Table 2 and Figure S1). Higher levels of several sphingomyelins (SMs), lysophosphatidylcholines (lysoPCs) and phosphatidylcholines (PCs) were observed in rs7903146 risk allele carriers (Table 2). After correction for multiple testing, there was no statistically significant genotype effect on any metabolite concentration in the fasting samples. 

**Table 2:** Selected metabolite concentrations (µmol/l) in the fasting state in carriers of TCF7L2 rs7903146 ( **CT**/**TT** genotype) and non-risk allele carriers (CC genotype).

	**CC genotype (n = 24)**	**CT/TT genotype (n = 17)**	**p-value**
C0 (µmol/l)	36.6 (6.6)	42.2 (9.8)	0.079
C2 (µmol/l)	7.9 (4.1)	8.12 (2.6)	0.914
C3 (µmol/l)	0.43 (0.14)	0.53 (0.20)	0.046
C4 (µmol/l)	0.21 (0.12)	0.34 (0.43)	0.039
C10 (µmol/l)	0.38 (0.15)	0.59 (0.54)	0.046
Non-essential amino acids (µmol/l)	1085.5 (186.5)	1226.6 (195.7)	0.018
Sum of glycine and serine (µmol/l)	293.2 (60.3)	325.6 (55.3)	0.088
Branched-chain amino acids (µmol/l)	515.6 (93.1)	550.1 (135.7)	0.177
Proline (µmol/l)	181.6 (39.9)	226.8 (57.8)	0.005
lysoPCs (µmol/l)	171.0 (52.2)	190.1 (38.2)	0.025
Saturated lysoPCs (µmol/l)	105.9 (24.1)	122.0 (21.7)	0.006
lysoPC a C14:0 (µmol/l)	6.2 (0.7)	6.6 (0.6)	0.032
lysoPC a C16:0 (µmol/l)	98.2 (23.4)	113.9 (21.4)	0.007
lysoPC a C20:4 (µmol/l)	6.5 (3.1)	7.44 (2.06)	0.021
PC ae C36:4 (µmol/l)	204.7 (47.6)	234.2 (59.2)	0.045
PC ae C38:4 (µmol/l)	13.7 (3.5)	15.0 (3.6)	0.029
PC ae C38:5 (µmol/l)	17.9 (4.7)	20.61 (5.2)	0.025
PC ae C40:5 (µmol/l)	3.2 (0.7)	3.5 (0.6)	0.038
PC ae C44:5(µmol/l)	1.6 (0.5)	1.9 (0.4)	0.008
PC ae C44:6(µmol/l)	1.0 (0.3)	1.2 (0.2)	0.030
SMs (µmol/l)	328.7 (57.8)	377.7 (100.9)	0.045
SM C (µmol/l)	284.5 (49.8)	327.5 (87.0)	0.045
Long SM-OH (µmol/l)	1.7 (0.4)	2.0 (0.7)	0.039
SM C16:0 (µmol/l)	121.5 (22.5)	141.6 (35.9)	0.023
SM-OH C22:1 (µmol/l)	17.4 (3.8)	20.0 (5.8)	0.049
SM-OH C24:1 (µmol/l)	1.7 (0.4)	2.0 (0.7)	0.039

Data are given as mean (standard deviation). Given are the crude p values before correction for multiple testing. Abbreviation of metabolites are explained in Table S1.

### Insulin response to intravenous glucose and insulin sensitivity

**Figure 1:**
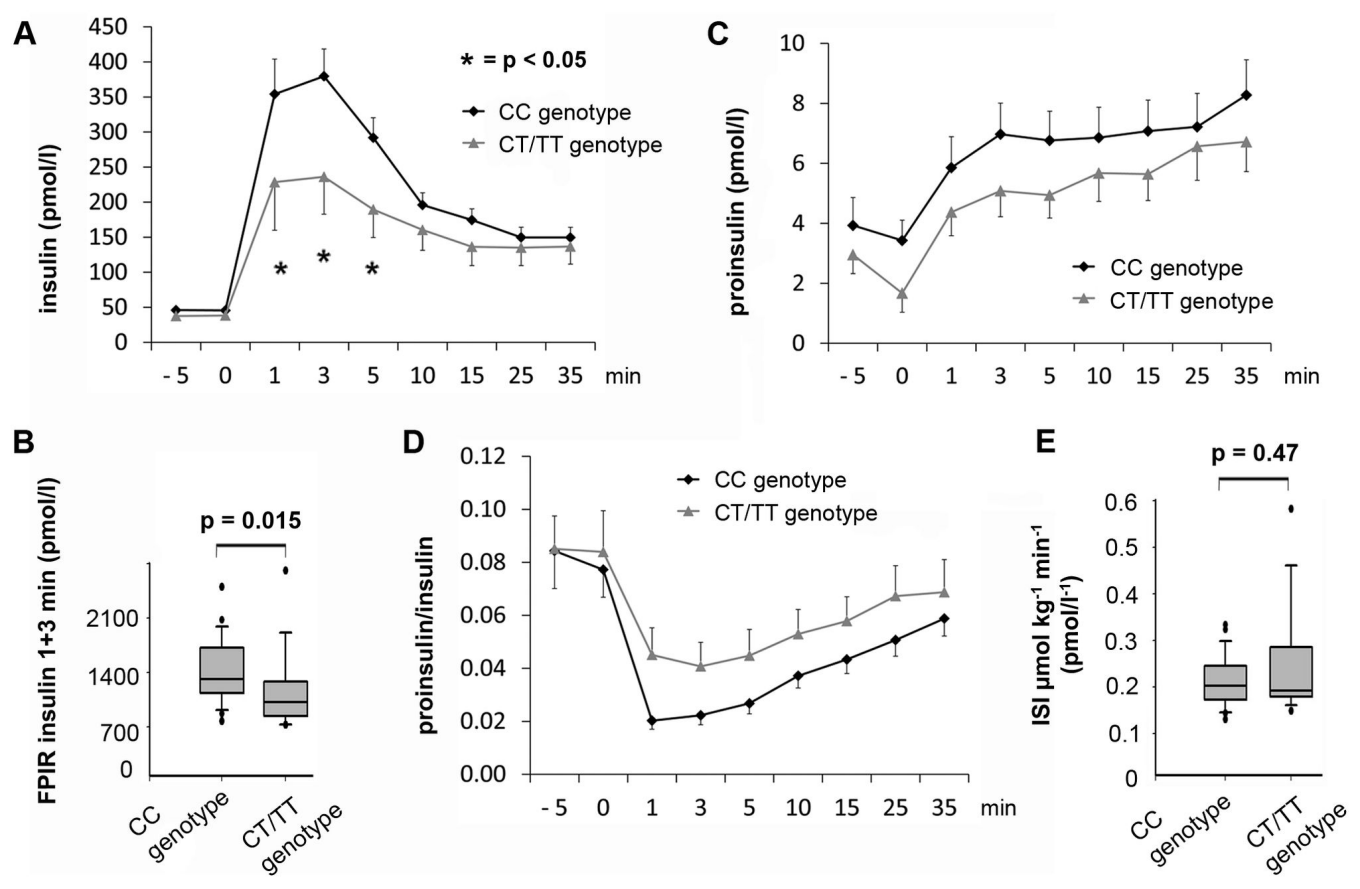
Response to an intravenous glucose challenge and insulin sensitivity as assessed by an EH clamp. (A) The insulin response was impaired at 1 (p = 0.03), 3 (p = 0.01) and 5 (p = 0.01) minutes after glucose challenge in the TCF7L2 group (CT/TT genotype) as compared to the control group (CC genotype). (B) The first phase insulin response (FPIR, sum of insulin values at 1 and 3 minutes) was significantly lower in TCF7L2 risk allele carriers. (C) TCF7L2 risk allele carriers had non-significantly lower proinsulin values after intravenous glucose challenge. (D) The proinsulin/insulin ratio was only slightly elevated in the TCF7L2 group. (E) ISI (insulin sensitivity index) displayed no significant difference in carriers of the TCF7L2 risk allele compared to controls. Data are given as mean and standard error of the mean in A, C and D.

The plasma insulin levels at 1, 3 and 5 minutes after the glucose injection and the first-phase insulin response (FPIR, sum of 1 + 3 minutes) were significantly reduced in carriers of the TCF7L2 risk allele compared to the control group (Figure 1A, 1B), whereas no significant differences in the second-phase insulin response were observed. There was a non-significant tendency towards lower proinsulin concentrations (Figure 1C) and increased proinsulin to insulin ratio in the TCF7L2 risk group after intravenous glucose injection (Figure 1D). The AUC of plasma glucose in the early and the late phase after the glucose bolus was similar in rs7903146 risk allele carriers and controls. No differences were found for insulin sensitivity as determined by insulin sensitivity index (ISI) during the EH clamp (Figure 1E). There was no significant difference in FPIR, second phase insulin response and insulin sensitivity between homozygous (TT) and heterozygous (CT) carriers of the TCF7L2 rs7903146 allele.

### Genotype effects on metabolite changes induced by ivGTT-EH clamp

**Figure 2:**
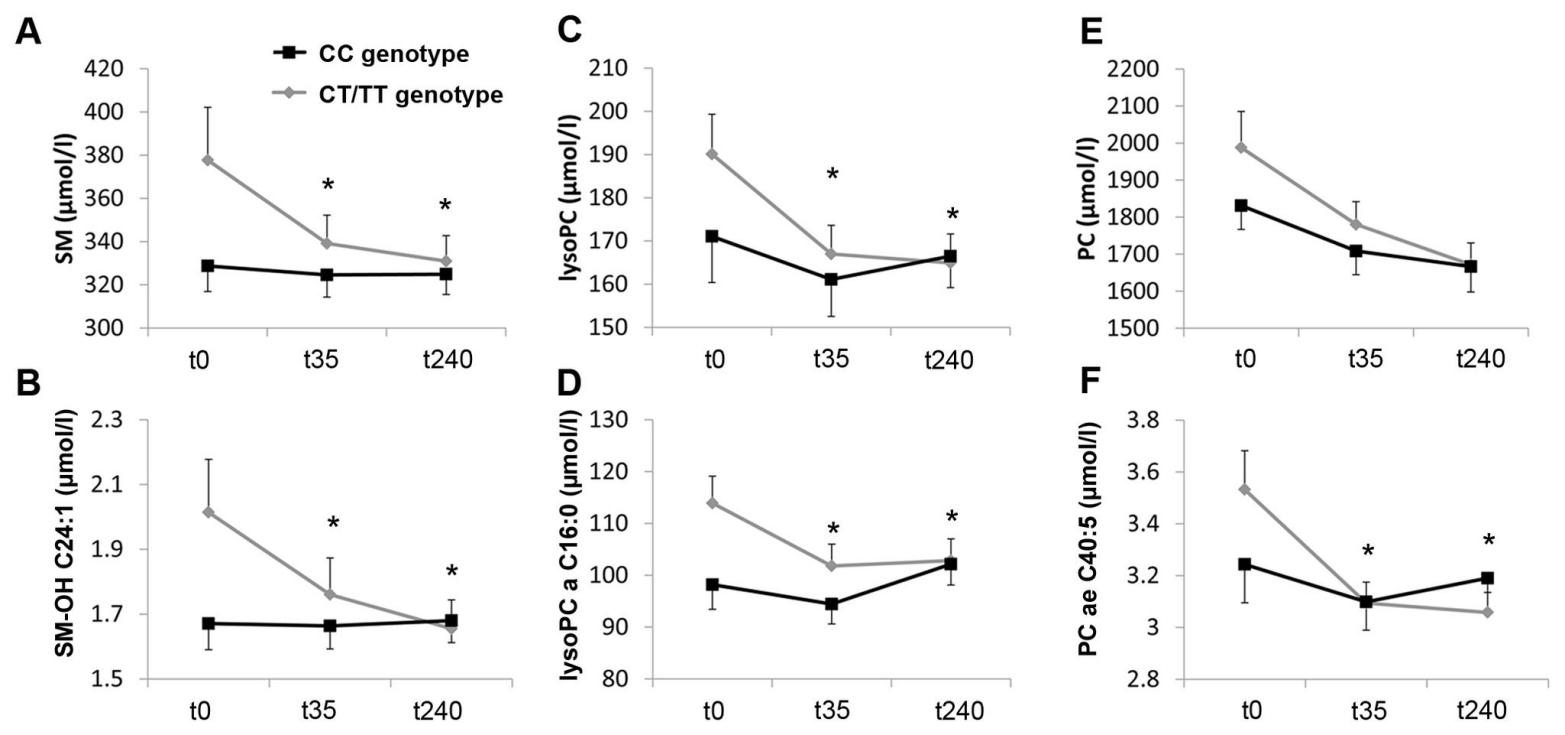
Metabolites displaying the highest differences between TCF7L2 risk genotype carriers and controls. Concentration of total plasma SM, lysoPC and PC levels and selected SM-, lysoPC-, and PC-species in the fasting state, 35 minutes after the intravenous glucose bolus (t35) and during the steady state of the EH clamp (t240). (A) A significantly higher decease of total SM plasma levels was observed in TCF7L2 rs7903146 allele carriers (CT/TT genotype) at t35 (p=3.5E-03) and t240 (p = 4.4E-03) compared to CC genotype carriers. (B) SM-OH C24:1 plasma levels displayed the strongest difference between the genotype groups in delta t35 (p = 8.3E-04) and delta t240 (p = 5.4E-04). (C) Delta t35 and delta t240 of total lysoPC was significantly different between the genotype groups (p = 3.0E-03 and p = 3.6E-03). (D) LysoPC a C16:0 presented higher delta t35 (p = 2.6E-03) and deltat240 (p = 7.9E-04) in the subjects carrying the TCF7L2 rs7903146 risk allele. (E) The sum of all PCs did not present significant differences. (F) However, several PC species decreased significantly stronger in risk allele carriers. Delta t35 (p = 2.7E-03) and t240 (p = 4.2E-03) of PC ae C40:5 are given as a representative example. Data are given as mean and standard error of the mean. * = p ≤ 0.05 after correction for multiple testing.

Metabolite concentrations were determined 35 minutes after glucose infusion (t35). Overall, 162 metabolite traits showed ivGTT-induced changes (p < 0.05) in both risk allele carriers or control subjects. For 17 metabolites, we observed a significant genotype effect on challenge-induced changes in TCF7L2 risk allele carriers compared to control subjects (Table 3). All differentially regulated plasma metabolites were phospholipids with the most pronounced differences observed in SMs. A significantly higher decline of metabolites (delta t35) was observed in 10 out of 13 SM species as well as for the sum of all SM (Figure 2A) and hydroxylated SM (SM-OH) species. Similar to SM levels, delta t35 for the sum of all measured lysoPC species (Figure 2C), saturated lysoPCs and of four out of 14 individual lysoPC species was significantly higher in TCF7/L2 risk allele carriers. Of 74 analysed PCs, only delta t35 of PC aa C28:1, PC aa C40:4 and PC ae C40:5 (Figure 2F) was significantly higher in the TCF7L2 group. Adjustment for fasting insulin or FPIR attenuated the results, suggesting that some alterations in phospholipids are influenced by disturbed insulin secretion (Table S2). 

**Table 3:** Metabolic traits with significant genotype effect in response to ivGTT (t0 versus t35).

	**Challenge response (iv glucose) CC genotype**			**Challenge response (iv glucose**)**CT/TT genotype**			**Genotype effect**			
Metabolite	β	se	p-value	β	se	p-value	β	se	p-value	p-value (corrected)
**Phosphatidycholines**										
PC aa C28:1	-0.13	0.11	2.40E-01	-0.6	0.13	1.40E-05	-0.47	0.17	7.30E-03	5.00E-02
PC aa C40:4	-0.05	0.08	5.60E-01	-0.48	0.10	8.40E-06	-0.43	0.13	1.80E-03	3.60E-02
PC ae C40:5	-0.1	0.10	4.90E-01	-0.84	0.18	1.10E-05	-0.74	0.24	2.70E-03	3.60E-02
**Lysophosphatidylcholines**										
lysoPC a C14:0	0.39	0.15	1.30E-02	-0.42	0.18	2.60E-02	-0.81	0.25	1.60E-03	3.60E-02
lysoPC a C16:0	-0.04	0.12	7.50E-01	-0.64	0.15	3.40E-05	-0.60	0.19	2.60E-03	3.60E-02
lysoPC a C16:1	-0.07	0.07	3.60E-01	-0.47	0.09	1.40E-06	-0.40	0.12	1.20E-03	3.60E-02
lysoPC a C17:0	-0.04	0.12	7.60E-01	-0.57	0.14	1.40E-04	-0.53	0.19	6.10E-03	4.70E-02
lysoPCs	-0.12	0.10	2.40E-01	-0.63	0.12	2.80E-06	-0.51	0.17	3.00E-03	3.60E-02
Saturated lysoPCs	-0.03	0.12	8.10E-01	-0.65	0.15	3.20E-05	-0.62	0.20	2.20E-03	3.60E-02
**Sphingomyelins**										
SM-OH C14:1	0.05	0.11	6.20E-01	-0.45	0.13	9.00E-04	-0.50	0.17	4.80E-03	3.90E-02
SM-OH C22:1	0.02	0.12	8.40E-01	-0.49	0.14	7.30E-04	-0.51	0.19	7.10E-03	5.00E-02
SM-OH C22:2	0.04	0.12	7.20E-01	-0.57	0.14	1.60E-04	-0.61	0.19	2.00E-03	3.60E-02
SM-OH C24:1	0.09	0.12	4.60E-01	-0.57	0.14	1.30E-04	-0.66	0.19	8.30E-04	3.60E-02
SM C16:0	0.06	0.15	7.00E-01	-0.64	0.18	5.70E-04	-0.70	0.24	4.30E-03	3.90E-02
SM C16:1	0.02	0.14	8.80E-01	-0.64	0.16	2.10E-04	-0.66	0.22	3.50E-03	3.60E-02
SM C18:0	0.00	0.12	9.90E-01	-0.57	0.15	2.10E-04	-0.57	0.19	4.70E-03	3.90E-02
SM C18:1	0.02	0.12	8.90E-01	-0.52	0.14	5.20E-04	-0.53	0.19	6.50E-03	4.80E-02
SM C24:0	0.05	0.13	6.80E-01	-0.61	0.16	2.70E-04	-0.67	0.21	2.60E-03	3.60E-02
SM C24:1	0.07	0.13	6.10E-01	-0.55	0.16	7.40E-04	-0.61	0.21	4.20E-03	3.90E-02
SMs	0.05	0.14	7.20E-01	-0.65	0.17	3.70E-04	-0.70	0.23	3.50E-03	3.60E-02
SM C	0.05	0.14	7.20E-01	-0.64	0.17	3.90E-04	-0.70	0.23	3.60E-03	3.60E-02
SM-OH	0.04	0.12	7.40E-01	-0.53	0.14	3.50E-04	-0.57	0.19	3.50E-03	3.60E-02
Long SMs	0.06	0.14	6.40E-01	-0.60	0.16	4.70E-04	-0.66	0.22	3.20E-03	3.60E-02
Long SM C	0.06	0.14	6.40E-01	-0.60	0.16	5.00E-04	-0.66	0.22	3.40E-03	3.60E-02
Long SM-OH	0.09	0.12	4.60E-01	-0.57	0.14	1.30E-04	-0.66	0.19	8.30E-04	3.60E-02

After the ivGTT, an EH clamp was performed inducing an anabolic state with high insulin plasma levels and increased uptake of glucose in peripheral organs. EH clamp was accompanied by a significant increase in carnitine (C0) levels in control subjects but not in TCF7L2 risk allele carriers and a marked decrease of short-chain acylcarnitines (C2, C3) and NEFAs levels in both groups (Figure S1). We observed a significant genotype effect at time-point t240 versus t0 in 17 metabolite traits (Table 4). Fasting insulin as well as FPIR modified genotype effects on metabolites (Table S3). The decrease of 8 SM species and the sum of all SMs were more pronounced in TCF7L2 risk allele carries and all but one SM-OH (SM-OH C14:1) were significantly altered. The most prominent down-regulation occurred in SM-OH C24:1 (Figure 2B). At t240, three lysoPC species (lysoPC a C16:0, lysoPC a C16:1, lysoPC a C17:0), the sum of all lysoPCs (Figure 2C) and the sum of saturated lysoPCs displayed a significant stronger decline in the TCF7L2 compared to the control group. Change in total PC levels was not different between genotype groups (Figure 2E). However, 7 unsaturated PC species decreased significantly stronger in the TCF7L2 group (PC aa C36:6, PC aa C40:4, PC ae C40:2, PC ae C40:5, PC ae C44:5, PC ae C44:6). 

**Table 4:** Metabolic traits with significant genotype effect in response to EH clamp (t0 versus t240).

	**Challenge response (clamp) CC genotype**			**Challenge response (clamp) CT/TT genotype**			**Genotype effect**			
Metabolite	β	se	p value	β	se	p value	β	se	p value	p value (corrected)
**Acylcarnitines**										
C0	0.66	0.14	1.10E-05	0.01	0.17	9.50E-01	-0.65	0.23	5.40E-03	3.80E-02
**Phosphatidylcholines**										
PC aa C36:6	-0.18	0.09	4.80E-02	-0.65	0.11	1.60E-07	-0.47	0.15	2.40E-03	3.80E-02
PC aa C40:4	-0.01	0.10	9.20E-01	-0.48	0.12	1.90E-04	-0.47	0.16	4.80E-03	3.80E-02
PC ae C40:2	-0.11	0.13	3.80E-01	-0.69	0.16	4.00E-05	-0.57	0.21	7.40E-03	4.70E-02
PC ae C40:5	0.02	0.18	9.10E-01	-0.84	0.22	2.80E-04	-0.86	0.29	4.20E-03	3.80E-02
PC ae C44:5	0.02	0.13	8.90E-01	-0.72	0.17	4.00E-05	-0.74	0.22	1.10E-03	2.70E-02
PC ae C44:6	-0.02	0.15	9.10E-01	-0.74	0.18	1.30E-04	-0.72	0.24	3.90E-03	3.80E-02
**Lysophosphatidylcholines**										
lysoPC a C16:0	0.33	0.16	3.90E-02	-0.56	0.19	4.70E-03	-0.88	0.25	7.90E-04	2.20E-02
lysoPC a C16:1	0.09	0.11	4.30E-01	-0.47	0.14	9.20E-04	-0.55	0.18	2.80E-03	3.80E-02
lysoPC a C17:0	0.24	0.14	7.80E-02	-0.56	0.17	1.10E-03	-0.81	0.22	4.50E-04	2.20E-02
lysoPCs	0.06	0.15	6.80E-01	-0.66	0.18	4.80E-04	-0.72	0.24	3.60E-03	3.80E-02
Saturated lysoPCs	0.33	0.16	3.90E-02	-0.57	0.19	4.30E-03	-0.90	0.26	7.40E-04	2.20E-02
**Sphingomyelins**										
SM-OH C16:1	0.00	0.12	9.90E-01	-0.52	0.14	4.50E-04	-0.52	0.19	6.90E-03	4.60E-02
SM-OH C22:1	0.04	0.13	7.50E-01	-0.62	0.16	1.90E-04	-0.66	0.21	2.30E-03	3.80E-02
SM-OH C22:2	0.13	0.13	3.30E-01	-0.62	0.16	2.20E-04	-0.75	0.21	7.00E-04	2.20E-02
SM-OH C24:1	0.10	0.13	4.60E-01	-0.68	0.16	7.80E-05	-0.78	0.21	5.40E-04	2.20E-02
SM C16:0	0.03	0.17	8.60E-01	-0.75	0.20	4.30E-04	-0.78	0.27	5.00E-03	3.80E-02
SM C16:1	-0.03	0.16	8.60E-01	-0.78	0.19	9.50E-05	-0.75	0.25	3.70E-03	3.80E-02
SM C18:1	0.04	0.14	7.70E-01	-0.6	0.17	5.80E-04	-0.64	0.22	4.90E-03	3.80E-02
SM C24:0	0.07	0.15	6.40E-01	-0.71	0.18	2.10E-04	-0.78	0.24	1.80E-03	3.70E-02
SMs	0.04	0.16	8.00E-01	-0.73	0.20	4.20E-04	-0.77	0.26	4.40E-03	3.80E-02
SM C	0.04	0.16	8.30E-01	-0.72	0.20	4.80E-04	-0.76	0.26	5.10E-03	3.80E-02
SM-OH	0.06	0.13	6.40E-01	-0.63	0.16	2.30E-04	-0.69	0.22	1.90E-03	3.70E-02
Long SMs	0.06	0.15	6.90E-01	-0.65	0.19	8.40E-04	-0.71	0.25	5.30E-03	3.80E-02
Long SM C	0.06	0.15	7.00E-01	-0.64	0.19	9.00E-04	-0.70	0.25	5.60E-03	3.90E-02
Long SM-OH	0.10	0.13	4.60E-01	-0.68	0.16	7.80E-05	-0.78	0.21	5.40E-04	2.20E-02

## Discussion

Using a targeted metabolomics platform, we identified alterations in sphingolipid, phosphatidylcholine and lysophosphatidylcholine metabolism in subjects at potentially increased risk for T2D defined by the presence of the rs7903146 risk genotype. The most important finding is that these differences were only observed after challenge tests and were present in a state when all conventional markers of glucose metabolism such as fasting and 2-h glucose in oGTT, fasting insulin, proinsulin and insulin sensitivity were still in the normal range.

Measurement of a large number of metabolites by targeted metabolomics is a promising approach to identify and quantify intermediate and endpoint compounds of metabolism related to specific disorders. This method has recently been successfully used to define metabolomic signatures of cardiovascular diseases, neurological disorders, obesity and diabetes mellitus [15,24–27]. Our hypothesis was that metabolic alterations may precede the development of glucose intolerance and that the disturbed metabolic homeostasis may be detected by differences in plasma metabolite concentrations. We chose to analyse non-diabetic male subjects harbouring the high risk allele TCF7L2 rs7903146 and compared them with age-, sex- and BMI-matched controls. Considering that TCF7L2 is involved in β-cell dysfunction including impaired insulin secretion and disturbed incretin effects as well as an enhanced rate of basal hepatic glucose production [3–7], changes of subsets of metabolites may provide novel insights into biochemical pathways involved in the development of T2D.

In our study we demonstrate the presence of impaired FPIR and normal insulin sensitivity in carriers of the TCF7L2 rs7903146 allele. Our data confirm the finding that TCF7L2 polymorphism is associated with an intrinsic β-cell dysfunction and/or a reduction of β-cell mass [4]. Incretins are thought to partly mediate the impaired insulin secretion in patients with TCF7L2 risk allele [28]. By challenge with intravenous glucose we can exclude any impact of incretins on metabolite concentrations in the present study. Marked differences in the regulation of plasma phospholipids were observed in ivGTT and EH clamp. A stronger reduction of plasma metabolite levels in TCF7L2 risk allele carriers compared to controls was observed for 8 SMs, 6 PCs and 3 lysoPCs (t0 versus t240). This finding may be explained by slightly increased basal metabolite levels at the starting point under fasting conditions and a stronger clearance of SMs, PCs and lysoPCs in the TCF7L2 group after glucose challenge and additional stimulation with high insulin levels. The fact that significant genotype effects were only observed in challenge tests may explain why other studies have not identified the same metabolite profiles in risk allele carriers [13,19]. 

The three major phospholipid groups (SMs, PCs, lysoPCs) are important structural components of plasma lipoproteins and cell membranes and are involved in regulation of cell function, membrane protein trafficking and inflammation [29]. In human plasma, PCs comprise about 60-70 %, SMs 10-20 % and lysoPCs 10-20 % of the circulating phospholipids. Increased plasma SM levels have been reported in subjects with subclinical atherosclerosis [30], coronary heart disease [31], obesity [32] and diabetes mellitus [13]. We did not detect significantly increased fasting SM levels in TCF7L2 risk allele carriers. The present study is the first investigating metabolic signatures after challenge tests in subjects with defined genetic risk for T2D. We observed a significant genotype effect in almost all SMs, suggesting a general alteration in the synthesis or breakdown of sphingolipid metabolism. Several studies indicate a role for SMs in the development of T2D by influencing insulin resistance as well as insulin secretion. In SM synthetase knockout mouse models it has been shown that the reduction of plasma membrane SMs increased insulin sensitivity [33,34]. Another study reported a decreased insulin secretion due to mitochondrial dysfunction as a consequence of altered membrane SM and ceramide contents [35]. Furthermore, apoptosis of β-cells is partly dependent on the action of sphingomyelinase degrading SMs to ceramide [36]. Inhibition of ceramide synthesis decreased β-cell apoptosis and defective protein trafficking in β-cells exposed to lipotoxicity [37]. Thus, ceramides derived from SM degradation may contribute to lipotoxicity-mediated β-cell apoptosis and may accelerate the observed reduction of FPIR in carriers of the TCF7L2 risk genotype. 

While plasma LDL- and HDL-cholesterol and triglyceride levels were similar in all probands, significant genotype effects were detected in long-chain lysoPCs (C16:0, C16:1, C17:0) and long-chain PC species (PCs containing C28-C44). The finding of a disturbed PC/lysoPC metabolism is in agreement with results from previous studies describing that altered PC and lysoPC plasma profiles are associated with T2D (increased PC aa (16:0/18:2); decreased PC aa (16:0/18:0), decreased PC aa (18:0/20:4), increased lysoPCs (C16:0, C18:0, C18:1, C18:2, C20:4)) [13], increased lysoPCs (C14:0, C16:1, C18:1, C20:5, C22:6) [38], impaired glucose tolerance (decreased lysoPC C18:2) ) [14,39] and increased risk for T2D (decreased PC ae (C34:3, C40:6, C42:5, C44:4, C44:5), increased PC aa (C32:1, C36:1, C38:3, C40:5)) [14]. In addition, increased levels of several lysoPCs were reported in subjects with obesity and insulin resistance [40]. Here, we did not find significantly increased basal lysoPC species in subjects at increased risk for T2D, but detected significant alterations in the regulation of PCs with fatty acid aliphatic tails between 36 and 44 carbons. This has not been described before in prediabetes or T2D and may indicate that in parallel with the modification of the long-chain lysoPCs there is also a disturbed regulation of very-long-chain PCs in risk subjects carrying the TCF7L2 risk allele. 

LysoPCs are produced by the action of phospholipase A2 (PLA2) or lecithin cholesterol acyltransferase (LCAT) on PCs and the activity of both enzymes is related with plasma lysoPC concentrations. LysoPCs, e.g. produced by increased PC hydrolysis during LDL oxidation, are strong pro-inflammatory lipid mediators [38] and are related to various pathophysiological conditions including endothelial dysfunction [41] and acute coronary syndrome [42]. Treatment of diabetic patients with metformin led to decreased lysoPC levels [43] and *in vitro* inhibition of PLA2 prevented palmitic acid-induced insulin resistance in L6 myotubes by reduced generation of lysoPC [44]. In contrast to these results, other studies demonstrated that lysoPCs stimulate glucose uptake in 3T3-L1 adipocytes by elevating glucose transporter type 4 levels at the plasma membrane yielding to lower blood glucose levels in normal and diabetic mice [45]. Treatment of cultured islet cells with phospholipase increased lysoPC contents and insulin secretion [46]. Thus, there may be positive and negative effects of some lysoPC species on insulin resistance and β-cell function. The significant genotype effects on lysoPCs containing C16:0, C16:1 and C17:0 fatty acid chains, which have been observed in the present study, may suggest an increased proinflammatory and proatherogenic state in TCF7L2 risk allele carriers. The underlying molecular mechanism in the regulation of circulating PC species remains unclear.

Our challenge tests revealed that changes in metabolic profiles of TCF7L2 risk allele carriers are reversible. Hyperinsulinemia in the EH-clamp was able to reduce the differences between the study groups. Although we did not observe any differences in basal glucose or insulin levels, adjustment for fasting insulin and FPIR attenuated the association of phospholipids in TCF7L2 risk carriers, suggesting that some alterations in metabolic homeostasis are dependent on disturbed insulin secretion, which can be overridden by high insulin doses. The most important sites for the action of insulin are liver, adipose tissue and muscle. Insulin inhibits gluconeogenesis in the liver, promotes the storage of glycogen and induces the cellular uptake of amino acids and fatty acids to produce proteins and lipids. Under high insulin concentrations there is a strong inhibition of lipolysis (e.g. reduction of NEFAs). One possible explanation for the decline of phospholipid species during the EH-clamp in TCF7L2 risk allele carriers is that the high insulin concentrations induce sphingomyelinase, down-regulate SM synthase or induce PLA2/LCAT, leading to decreasing plasma concentration of SMs, lysoPCs and PCs. The exact mechanisms by which high insulin concentrations influence phosholipid synthesis or degradation are incompletely understood and require further experimental and clinical studies. 

In contrast to other studies indicating an association of increased plasma levels of several amino acids with prediabetes and T2D [14,27,47], we did not find significant changes in plasma amino acids. Different amino acid concentrations have been shown to be associated with insulin resistance in obese patients [24,48]. One study reported that elevated plasma levels of three branched-chain amino acids (valine, leucine and isoleucine) and two aromatic amino acids (tyrosine and phenylalanine) are correlated with future risk for T2D [27]. In the present study we did not observe any difference in amino acid levels, which may be explained by the fact that our study population was not obese and had normal insulin sensitivity. In addition, only some probands carrying the risk allele may progress to overt T2D. It may be speculated that changes in amino acids occur at a late stage during the development of T2D [39].

Limitations of the present study are the moderate sample size, the lack of prospective follow-up investigation and the complex interaction between the components of glucose, lipid and protein metabolism, which may limit the interpretation of the results. It is obvious that our findings need to be confirmed in other independent populations. We are not able to make any causal statements on the role of metabolic profiles on future development of T2D. We used a targeted approach focussing mainly on lipids. Thus, we cannot exclude associations between TCF7L2 genotype and other metabolic products of carbohydrate or protein metabolism, e.g. metabolites from the tricarboxylic acid cycle. In addition, the exact biochemical mechanisms leading to changes of these metabolites remain unknown and the clarification of the biological significance of these findings requires further investigation.

In conclusion, this study provides evidence for a complex perturbation of phospholipid metabolism possibly linked to early β-cell dysfunction in TCF7L2 risk allele carriers, in a state when conventional parameters of glucose homeostasis are not yet affected. Our findings suggest subtle modifications in the regulation of intermediate metabolism, which can only be detected by metabolic challenge tests. These data may contribute to a better understanding of the biochemical networks underlying the development of T2D in subject with the TCF7L2 risk genotype. 

## Supporting Information

Figure S1 **Change of NEFAs, selected acylcarnitines and amino acid plasma levels during challenge tests.** Concentrations are given in the fasting state, 35 minutes after the intravenous glucose bolus (t35) and at the steady state of the EH clamp (t240). (A) Fasting NEFA plasma concentrations dropped in both groups to similar levels during the challenge test. (B) The increase of C0 plasma levels was more pronounced in the control group. (C) Plasma C2 decreased in both groups in the EH clamp. (D) C4 levels were higher in the fasting state in TCF7L2 risk allele carriers (CT/TT genotype), which was not significant after correction for multiple testing. During ivGTT/EH-clamp C4 levels decreased to similar levels in both groups. (E) The sum of the branched-amino acids valine, leucine and isoleucine as well as (F) the sum of the gluconeogenic amino acids serine and glycine decreased similarly in both groups.
(TIF)

Table S1 **List of all determined plasma metabolites.**
(DOC)

Table S2 **Genotype effects in response to ivGTT (t0 versus t35) on selected metabolites from Table 3 after adjustment for fasting insulin or FPIR.**
(DOC)

Table S3 **Genotype effects in response to EH clamp (t0 versus t240) on selected metabolites from Table 4 after adjustment for fasting insulin or FPIR.**
(DOC)
