# Supplementary figures and images for: Plasma Metabolomics Reveal Alterations of Sphingo- and Glycerophospholipid Levels in Non-Diabetic Carriers of the Transcription Factor 7-Like 2 Polymorphism rs7903146
Source: PLoS One. 2013 Oct 24;8(10):e78430. doi: 10.1371/journal.pone.0078430 (PMC3813438; doi:10.1371/journal.pone.0078430)

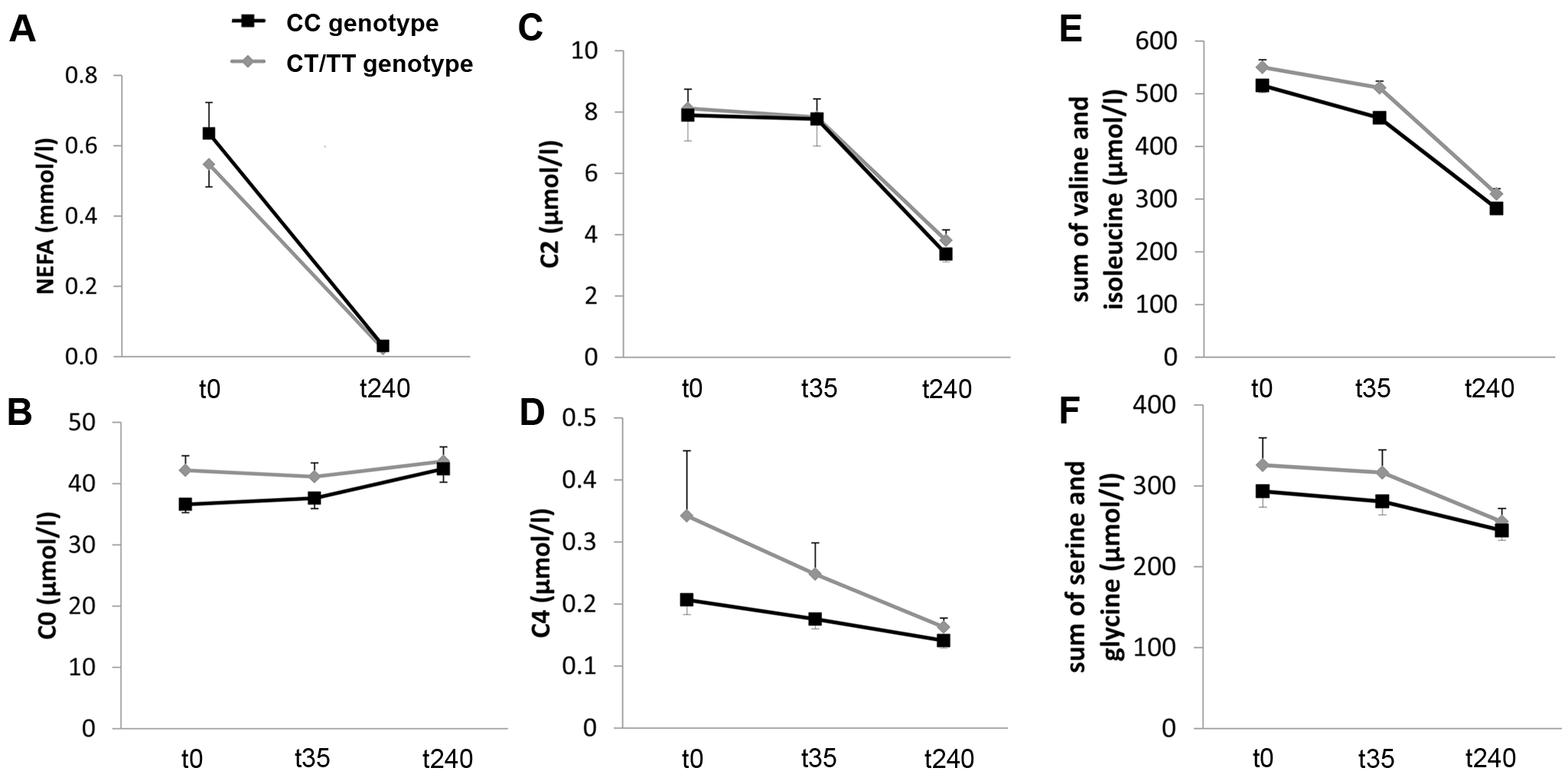

Supplement: Figure S1 — Change of NEFAs, selected acylcarnitines and amino acid plasma levels during challenge tests. Concentrations are given in the fasting state, 35 minutes after the intravenous glucose bolus (t35) and at the steady state of the EH clamp (t240). (A) Fasting NEFA plasma concentrations dropped in both groups to similar levels during the challenge test. (B) The increase of C0 plasma levels was more pronounced in the control group. (C) Plasma C2 decreased in both groups in the EH clamp. (D) C4 levels were higher in the fasting state in TCF7L2 risk allele carriers (CT/TT genotype), which was not significant after correction for multiple testing. During ivGTT/EH-clamp C4 levels decreased to similar levels in both groups. (E) The sum of the branched-amino acids valine, leucine and isoleucine as well as (F) the sum of the gluconeogenic amino acids serine and glycine decreased similarly in both groups. (TIF) [file pone.0078430.s001.tif]
