# Supplementary material for: Plasma Metabolomics Reveal Alterations of Sphingo- and Glycerophospholipid Levels in Non-Diabetic Carriers of the Transcription Factor 7-Like 2 Polymorphism rs7903146
Source: PLoS One. 2013 Oct 24;8(10):e78430. doi: 10.1371/journal.pone.0078430 (PMC3813438; doi:10.1371/journal.pone.0078430)
Supplement: Table S1 — List of all determined plasma metabolites. (DOC) [file pone.0078430.s002.doc]

**Table S**1

| **Abbreviation** | **Full biochemical name** | **Passed quality control** |
| --- | --- | --- |
| **Acylcarnitines** | | |
| **C0** | DL-Carnitine | yes |
| **C2** | Acetyl-L-carnitine | yes |
| **C3** | Propionyl-L-carnitine | yes |
| **C3:1** | Propenyl-L-carnitine | no |
| **C3-DC/ C4-OH** | Malonyl-L-carnitine / Hydroxybutyryl-L-carnitine | no |
| **C3-DC-M / C5-OH** | Methylmalonyl-L-carnitine / Hydroxyvaleryl-L-carnitine | no |
| **C3-OH** | Hydroxypropionyl-L-carnitine | no |
| **C4** | Butyryl-L-carnitine | yes |
| **C4:1** | Butenyl-L-carnitine | no |
| **C4:1-DC/ C6** | Fumaryl-L-carnitine / Hexanoyl-L-carnitine | yes |
| **C5** | Valeryl-L-carnitine | no |
| **C5:1** | Tiglyl-L-carnitine | yes |
| **C5:1-DC** | Glutaconyl-L-carnitine | no |
| **C5-DC/ C6-OH** | Glutaryl-L-carnitine / Hydroxyhexanoyl-L-carnitine | no |
| **C5-M-DC** | Methylglutaryl-L-carnitine | no |
| **C6:1** | Hexenoyl-L-carnitine | no |
| **C7-DC** | Pimelyl-L-carnitine | yes |
| **C8** | Octanoyl-L-carnitine | yes |
| **C8:1** | Octenoyl-L-carnitine | yes |
| **C9** | Nonayl-L-carnitine | no |
| **C10** | Decanoyl-L-carnitine | yes |
| **C10:1** | Decenoyl-L-carnitine | yes |
| **C10:2** | Decadienyl-L-carnitine | yes |
| **C12** | Dodecanoyl-L-carnitine | yes |
| **C12:1** | Dodecenoyl-L-carnitine | yes |
| **C12-DC** | Dodecanedioyl-L-carnitine | no |
| **C14** | Tetradecanoyl-L-carnitine | yes |
| **C14:1** | Tetradecenoyl-L-carnitine | yes |
| **C14:1-OH** | Hydroxytetradecenoyl-L-carnitine | no |
| **C14:2** | Tetradecadienyl-L-carnitine | no |
| **C14:2-OH** | Hydroxytetradecadienyl-L-carnitine | no |
| **C16** | Hexadecanoyl-L-carnitine | yes |
| **C16:1** | Hexadecenoyl-L-carnitine | yes |
| **C16:1-OH** | Hydroxyhexadecenoyl-L-carnitine | no |
| **C16:2** | Hexadecadienyl-L-carnitine | no |
| **C16:2-OH** | Hydroxyhexadecadienyl-L-carnitine | no |
| **C16-OH** | Hydroxyhexadecanoyl-L-carnitine | no |
| **C18** | Octadecanoyl-L-carnitine | yes |
| **C18:1** | Octadecenoyl-L-carnitine | yes |
| **C18:1-OH** | Hydroxyoctadecenoyl-L-carnitine | no |
| **C18:2** | Octadecadienyl-L-carnitine | yes |
| **Sugars** | | |
| **H1** | Hexose | yes |
| **Amino acids** | | |
| **Arg** | Arginine | yes |
| **Gln** | Glutamine | yes |
| **Gly** | Glycine | yes |
| **His** | Histidine | yes |
| **Met** | Methionine | yes |
| **Orn** | Ornithine | yes |
| **Phe** | Phenylalanine | yes |
| **Pro** | Proline | yes |
| **Ser** | Serine | yes |
| **Thr** | Threonine | yes |
| **Trp** | Tryptophan | yes |
| **Tyr** | Tyrosine | yes |
| **Val** | Valine | yes |
| **xLeu** | Leucine/Isoleucine | yes |
| **Diacyl phosphatidylcholines** | | |
| **PC aa C24:0** | Phosphatidylcholine diacyl C24:0 | no |
| **PC aa C26:0** | Phosphatidylcholine diacyl C26:0 | no |
| **PC aa C28:1** | Phosphatidylcholine diacyl C28:1 | yes |
| **PC aa C30:0** | Phosphatidylcholine diacyl C30:0 | yes |
| **PC aa C30:2** | Phosphatidylcholine diacyl C30:2 | no |
| **PC aa C32:0** | Phosphatidylcholine diacyl C32:0 | yes |
| **PC aa C32:1** | Phosphatidylcholine diacyl C32:1 | yes |
| **PC aa C32:2** | Phosphatidylcholine diacyl C32:2 | yes |
| **PC aa C32:3** | Phosphatidylcholine diacyl C32:3 | yes |
| **PC aa C34:1** | Phosphatidylcholine diacyl C34:1 | yes |
| **PC aa C34:2** | Phosphatidylcholine diacyl C34:2 | yes |
| **PC aa C34:3** | Phosphatidylcholine diacyl C34:3 | yes |
| **PC aa C34:4** | Phosphatidylcholine diacyl C34:4 | yes |
| **PC aa C36:0** | Phosphatidylcholine diacyl C36:0 | yes |
| **PC aa C36:1** | Phosphatidylcholine diacyl C36:1 | yes |
| **PC aa C36:2** | Phosphatidylcholine diacyl C36:2 | yes |
| **PC aa C36:3** | Phosphatidylcholine diacyl C36:3 | yes |
| **PC aa C36:4** | Phosphatidylcholine diacyl C36:4 | yes |
| **PC aa C36:5** | Phosphatidylcholine diacyl C36:5 | yes |
| **PC aa C36:6** | Phosphatidylcholine diacyl C36:6 | yes |
| **PC aa C38:0** | Phosphatidylcholine diacyl C38:0 | yes |
| **PC aa C38:1** | Phosphatidylcholine diacyl C38:1 | yes |
| **PC aa C38:3** | Phosphatidylcholine diacyl C38:3 | yes |
| **PC aa C38:4** | Phosphatidylcholine diacyl C38:4 | yes |
| **PC aa C38:5** | Phosphatidylcholine diacyl C38:5 | yes |
| **PC aa C38:6** | Phosphatidylcholine diacyl C38:6 | yes |
| **PC aa C40:1** | Phosphatidylcholine diacyl C40:1 | yes |
| **PC aa C40:2** | Phosphatidylcholine diacyl C40:2 | no |
| **PC aa C40:3** | Phosphatidylcholine diacyl C40:3 | yes |
| **PC aa C40:4** | Phosphatidylcholine diacyl C40:4 | yes |
| **PC aa C40:5** | Phosphatidylcholine diacyl C40:5 | yes |
| **PC aa C40:6** | Phosphatidylcholine diacyl C40:6 | yes |
| **PC aa C42:0** | Phosphatidylcholine diacyl C42:0 | yes |
| **PC aa C42:1** | Phosphatidylcholine diacyl C42:1 | yes |
| **PC aa C42:2** | Phosphatidylcholine diacyl C42:2 | yes |
| **PC aa C42:4** | Phosphatidylcholine diacyl C42:4 | yes |
| **PC aa C42:5** | Phosphatidylcholine diacyl C42:5 | yes |
| **PC aa C42:6** | Phosphatidylcholine diacyl C42:6 | yes |
| **Acyl-alkyl phosphatidylcholines** | | |
| **PC ae C30:0** | Phosphatidylcholine acyl-alkyl C30:0 | yes |
| **PC ae C30:1** | Phosphatidylcholine acyl-alkyl C30:1 | no |
| **PC ae C30:2** | Phosphatidylcholine acyl-alkyl C30:2 | no |
| **PC ae C32:1** | Phosphatidylcholine acyl-alkyl C32:1 | yes |
| **PC ae C32:2** | Phosphatidylcholine acyl-alkyl C32:2 | yes |
| **PC ae C34:0** | Phosphatidylcholine acyl-alkyl C34:0 | yes |
| **PC ae C34:1** | Phosphatidylcholine acyl-alkyl C34:1 | yes |
| **PC ae C34:2** | Phosphatidylcholine acyl-alkyl C34:2 | yes |
| **PC ae C34:3** | Phosphatidylcholine acyl-alkyl C34:3 | yes |
| **PC ae C36:0** | Phosphatidylcholine acyl-alkyl C36:0 | yes |
| **PC ae C36:1** | Phosphatidylcholine acyl-alkyl C36:1 | yes |
| **PC ae C36:2** | Phosphatidylcholine acyl-alkyl C36:2 | yes |
| **PC ae C36:3** | Phosphatidylcholine acyl-alkyl C36:3 | yes |
| **PC ae C36:4** | Phosphatidylcholine acyl-alkyl C36:4 | yes |
| **PC ae C36:5** | Phosphatidylcholine acyl-alkyl C36:5 | yes |
| **PC ae C38:0** | Phosphatidylcholine acyl-alkyl C38:0 | yes |
| **PC ae C38:1** | Phosphatidylcholine acyl-alkyl C38:1 | yes |
| **PC ae C38:2** | Phosphatidylcholine acyl-alkyl C38:2 | yes |
| **PC ae C38:3** | Phosphatidylcholine acyl-alkyl C38:3 | yes |
| **PC ae C38:4** | Phosphatidylcholine acyl-alkyl C38:4 | yes |
| **PC ae C38:5** | Phosphatidylcholine acyl-alkyl C38:5 | yes |
| **PC ae C38:6** | Phosphatidylcholine acyl-alkyl C38:6 | yes |
| **PC ae C40:0** | Phosphatidylcholine acyl-alkyl C40:0 | no |
| **PC ae C40:1** | Phosphatidylcholine acyl-alkyl C40:1 | yes |
| **PC ae C40:2** | Phosphatidylcholine acyl-alkyl C40:2 | yes |
| **PC ae C40:3** | Phosphatidylcholine acyl-alkyl C40:3 | yes |
| **PC ae C40:4** | Phosphatidylcholine acyl-alkyl C40:4 | yes |
| **PC ae C40:5** | Phosphatidylcholine acyl-alkyl C40:5 | yes |
| **PC ae C40:6** | Phosphatidylcholine acyl-alkyl C40:6 | no |
| **PC ae C42:0** | Phosphatidylcholine acyl-alkyl C42:0 | yes |
| **PC ae C42:1** | Phosphatidylcholine acyl-alkyl C42:1 | yes |
| **PC ae C42:2** | Phosphatidylcholine acyl-alkyl C42:2 | yes |
| **PC ae C42:3** | Phosphatidylcholine acyl-alkyl C42:3 | yes |
| **PC ae C42:4** | Phosphatidylcholine acyl-alkyl C42:4 | yes |
| **PC ae C42:5** | Phosphatidylcholine acyl-alkyl C42:5 | yes |
| **PC ae C44:3** | Phosphatidylcholine acyl-alkyl C44:3 | yes |
| **PC ae C44:4** | Phosphatidylcholine acyl-alkyl C44:4 | yes |
| **PC ae C44:5** | Phosphatidylcholine acyl-alkyl C44:5 | yes |
| **PC ae C44:6** | Phosphatidylcholine acyl-alkyl C44:6 | yes |
| **Lysophosphatidylcholines** | | |
| **lysoPC a C6:0** | Lysophosphatidylcholine acyl C6:0 | no |
| **lysoPC a C14:0** | Lysophosphatidylcholine acyl C14:0 | yes |
| **lysoPC a C16:0** | Lysophosphatidylcholine acyl C16:0 | yes |
| **lysoPC a C16:1** | Lysophosphatidylcholine acyl C16:1 | yes |
| **lysoPC a C17:0** | Lysophosphatidylcholine acyl C17:0 | yes |
| **lysoPC a C18:0** | Lysophosphatidylcholine acyl C18:0 | no |
| **lysoPC a C18:1** | Lysophosphatidylcholine acyl C18:1 | yes |
| **lysoPC a C18:2** | Lysophosphatidylcholine acyl C18:2 | yes |
| **lysoPC a C20:3** | Lysophosphatidylcholine acyl C20:3 | yes |
| **lysoPC a C20:4** | Lysophosphatidylcholine acyl C20:4 | yes |
| **lysoPC a C24:0** | Lysophosphatidylcholine acyl C24:0 | no |
| **lysoPC a C26:0** | Lysophosphatidylcholine acyl C26:0 | no |
| **lysoPC a C26:1** | Lysophosphatidylcholine acyl C26:1 | yes |
| **lysoPC a C28:0** | Lysophosphatidylcholine acyl C28:0 | no |
| **lysoPC a C28:1** | Lysophosphatidylcholine acyl C28:1 | no |
| **Sphingomyelines** | | |
| **SM-OH C14:1** | Hydroxysphingomyeline C14:1 | yes |
| **SM-OH C16:1** | Hydroxysphingomyeline C16:1 | yes |
| **SM-OH C22:1** | Hydroxysphingomyeline C22:1 | yes |
| **SM-OH C22:2** | Hydroxysphingomyeline C22:2 | yes |
| **SM-OH C24:1** | Hydroxysphingomyeline C24:1 | yes |
| **SM C16:0** | Sphingomyeline C16:0 | yes |
| **SM C16:1** | Sphingomyeline C16:1 | yes |
| **SM C18:0** | Sphingomyeline C18:0 | yes |
| **SM C18:1** | Sphingomyeline C18:1 | yes |
| **SM C20:2** | Sphingomyeline C20:2 | yes |
| **SM C22:3** | Sphingomyeline C22:3 | no |
| **SM C24:0** | Sphingomyeline C24:0 | yes |
| **SM C24:1** | Sphingomyeline C24:1 | yes |
| **SM C26:0** | Sphingomyeline C26:0 | no |
| **SM C26:1** | Sphingomyeline C26:1 | no |
