# Supplementary material for: Plasma Metabolomics Reveal Alterations of Sphingo- and Glycerophospholipid Levels in Non-Diabetic Carriers of the Transcription Factor 7-Like 2 Polymorphism rs7903146
Source: PLoS One. 2013 Oct 24;8(10):e78430. doi: 10.1371/journal.pone.0078430 (PMC3813438; doi:10.1371/journal.pone.0078430)
Supplement: Table S2 — Genotype effects in response to ivGTT (t0 versus t35) on selected metabolites from Table 3 after adjustment for fasting insulin or FPIR. (DOC) [file pone.0078430.s003.doc]

**Table S2**

|  | **Original model** | | **+ FPIR** | | **+ Fasting insulin** | |
| --- | --- | --- | --- | --- | --- | --- |
| **Metabolite** | **β** | **p-value** | **β** | **p-value** | **β** | **p-value** |
| **Phosphatidycholines** | | | | | | |
| PC aa C28:1 | -0.47 | 7.30E-03 | -0.24 | 0.032 | -0.25 | 0.027 |
| PC aa C40:4 | -0.43 | 1.80E-03 | -0.21 | 0.014 | -0.21 | 0.017 |
| PC ae C40:5 | -0.74 | 2.70E-03 | -0.34 | 0.027 | -0.34 | 0.028 |
| **Lysophosphatidylcholines** | | | | | | |
| lysoPC a C14:0 | -0.81 | 1.60E-03 | -0.43 | 0.007 | -0.42 | 0.010 |
| lysoPC a C16:0 | -0.6 | 2.60E-03 | -0.27 | 0.034 | -0.27 | 0.034 |
| lysoPC a C16:1 | -0.4 | 1.20E-03 | -0.19 | 0.017 | -0.18 | 0.020 |
| lysoPC a C17:0 | -0.53 | 6.10E-03 | -0.24 | 0.047 | -0.24 | 0.054 |
| lysoPCs | -0.51 | 3.00E-03 | -0.23 | 0.032 | -0.24 | 0.032 |
| Saturated lysoPCs | -0.62 | 2.20E-03 | -0.28 | 0.030 | -0.28 | 0.031 |
| **Sphingomyelins** | | | | | | |
| SM (OH) C14:1 | -0.5 | 4.80E-03 | -0.24 | 0.035 | -0.26 | 0.025 |
| SM (OH) C22:1 | -0.51 | 7.10E-03 | -0.23 | 0.055 | -0.23 | 0.058 |
| SM (OH) C22:2 | -0.61 | 2.00E-03 | -0.28 | 0.028 | -0.28 | 0.027 |
| SM (OH) C24:1 | -0.66 | 8.30E-04 | -0.31 | 0.016 | -0.32 | 0.014 |
| SM C16:0 | -0.7 | 4.30E-03 | -0.34 | 0.030 | -0.35 | 0.025 |
| SM C16:1 | -0.66 | 3.50E-03 | -0.31 | 0.030 | -0.31 | 0.033 |
| SM C18:0 | -0.57 | 4.70E-03 | -0.26 | 0.040 | -0.27 | 0.039 |
| SM C18:1 | -0.53 | 6.50E-03 | -0.24 | 0.048 | -0.25 | 0.045 |
| SM C24:0 | -0.67 | 2.60E-03 | -0.32 | 0.023 | -0.32 | 0.024 |
| SM C24:1 | -0.61 | 4.20E-03 | -0.28 | 0.040 | -0.27 | 0.047 |
| SMs | -0.7 | 3.50E-03 | -0.33 | 0.031 | -0.33 | 0.030 |
| SM C | -0.7 | 3.60E-03 | -0.33 | 0.031 | -0.33 | 0.030 |
| SM-OH | -0.57 | 3.50E-03 | -0.26 | 0.034 | -0.27 | 0.034 |
| Long SMs | -0.66 | 3.20E-03 | -0.31 | 0.032 | -0.30 | 0.037 |
| Long SM C | -0.66 | 3.40E-03 | -0.31 | 0.033 | -0.30 | 0.038 |
| Long SM-OH | -0.66 | 8.30E-04 | -0.31 | 0.016 | -0.32 | 0.014 |
