# Supplementary material for: Plasma Metabolomics Reveal Alterations of Sphingo- and Glycerophospholipid Levels in Non-Diabetic Carriers of the Transcription Factor 7-Like 2 Polymorphism rs7903146
Source: PLoS One. 2013 Oct 24;8(10):e78430. doi: 10.1371/journal.pone.0078430 (PMC3813438; doi:10.1371/journal.pone.0078430)
Supplement: Table S3 — Genotype effects in response to EH clamp (t0 versus t240) on selected metabolites from Table 4 after adjustment for fasting insulin or FPIR. (DOC) [file pone.0078430.s004.doc]

**Table S3**

|  | **Original model** | | **+ FPIR** | | **+ Fasting insulin** | |
| --- | --- | --- | --- | --- | --- | --- |
| **Metabolite** | **β** | **p-value** | **β** | **p-value** | **β** | **p-value** |
| **Acylcarnitines** | | | | | | |
| C0 | -0.65 | 5.40E-03 | -0.33 | 0.022 | -0.35 | 0.019 |
| **Phosphatidylcholines** | | | | | | |
| PC aa C36:6 | -0.47 | 2.40E-03 | -0.26 | 0.007 | -0.25 | 0.008 |
| PC aa C40:4 | -0.47 | 4.80E-03 | -0.26 | 0.012 | -0.26 | 0.011 |
| PC ae C40:2 | -0.57 | 7.40E-03 | -0.31 | 0.020 | -0.32 | 0.017 |
| PC ae C40:5 | -0.86 | 4.20E-03 | -0.48 | 0.011 | -0.49 | 0.009 |
| PC ae C44:5 | -0.74 | 1.10E-03 | -0.41 | 0.004 | -0.43 | 0.002 |
| PC ae C44:6 | -0.72 | 3.90E-03 | -0.37 | 0.018 | -0.40 | 0.011 |
| **Lysophosphatidylcholines** | | | | | | |
| lysoPC a C16:0 | -0.88 | 7.90E-04 | -0.49 | 0.003 | -0.47 | 0.004 |
| lysoPC a C16:1 | -0.55 | 2.80E-03 | -0.32 | 0.005 | -0.30 | 0.009 |
| lysoPC a C17:0 | -0.81 | 4.50E-04 | -0.40 | 0.006 | -0.39 | 0.008 |
| lysoPCs | -0.72 | 3.60E-03 | -0.39 | 0.010 | -0.38 | 0.012 |
| Saturated lysoPCs | -0.9 | 7.40E-04 | -0.49 | 0.003 | -0.48 | 0.004 |
| **Sphingomyelins** | | | | | | |
| SM (OH) C16:1 | -0.52 | 6.90E-03 | -0.29 | 0.016 | -0.31 | 0.010 |
| SM (OH) C22:1 | -0.66 | 2.30E-03 | -0.34 | 0.012 | -0.35 | 0.010 |
| SM (OH) C22:2 | -0.75 | 7.00E-04 | -0.37 | 0.008 | -0.38 | 0.007 |
| SM (OH) C24:1 | -0.78 | 5.40E-04 | -0.43 | 0.002 | -0.44 | 0.002 |
| SM C16:0 | -0.78 | 5.00E-03 | -0.41 | 0.017 | -0.44 | 0.011 |
| SM C16:1 | -0.75 | 3.70E-03 | -0.41 | 0.011 | -0.43 | 0.008 |
| SM C18:1 | -0.64 | 4.90E-03 | -0.35 | 0.014 | -0.37 | 0.009 |
| SM C24:0 | -0.78 | 1.80E-03 | -0.42 | 0.008 | -0.43 | 0.006 |
| SMs | -0.77 | 4.40E-03 | -0.41 | 0.016 | -0.43 | 0.012 |
| SM C | -0.76 | 5.10E-03 | -0.41 | 0.017 | -0.42 | 0.013 |
| SM-OH | -0.69 | 1.90E-03 | -0.36 | 0.011 | -0.37 | 0.009 |
| Long SMs | -0.71 | 5.30E-03 | -0.37 | 0.020 | -0.38 | 0.019 |
| Long SM C | -0.7 | 5.60E-03 | -0.37 | 0.021 | -0.37 | 0.020 |
| Long SM-OH | -0.78 | 5.40E-04 | -0.43 | 0.002 | -0.44 | 0.002 |
